# Divergent c-MYC Expression Patterns in NET and NEC: Insights from a Multicentre Cohort of 1380 Neuroendocrine Neoplasms

**DOI:** 10.1007/s12022-026-09935-x

**Published:** 2026-09-12

**Authors:** Maxime Schmitt, Katharina Ofner, Detlef Klaus Bartsch, Daniel-Christoph Wagner, Anja Rinke, Thomas Gress, Marcus Kremer, Matthias Evert, Nic G Reitsam, Viktorie Koberova, Bruno Märkl, Alexander Quaas, Markus Eckstein, Konrad Steinestel, Axel Pagenstecher, Ursula Keber, Carsten Denkert, Katja Steiger, Sebastian Foersch, Günter Klöppel, Atsuko Kasajima, Hanibal Bohnenberger, Moritz Jesinghaus

**Affiliations:** 1 Institute of Pathology, Marburg University and University Medical Centre Marburg, Baldingerstrasse, Marburg, 35043 Germany; 2 Institute of Pathology, University Medical Centre Göttingen, Göttingen, Germany; 3 Department of Visceral-, Thoracic- and Vascular-Surgery, Marburg University and University Medical Centre Marburg, Marburg, Germany; 4 Institute of Pathology, University Medical Centre Mainz, Mainz, Germany; 5 Department of Gastroenterology, Endocrinology and Infectious Diseases, Marburg University and University Medical Centre Marburg, Marburg, Germany; 6 Institute of Pathology, Städtisches Klinikum München, Munich, Germany; 7 Institute of Pathology, University Medical Centre Regensburg, Regensburg, Germany; 8 Pathology, Faculty of Medicine, University of Augsburg, Augsburg, Germany; 9 Bavarian Cancer Research Centre (BKFZ), Augsburg, Germany; 10 Institute of Pathology, University Medical Centre Cologne, Cologne, Germany; 11 Institute of Pathology, University Medical Centre Erlangen, Friedrich-Alexander-University Erlangen-Nuremberg, Erlangen, Germany; 12 Institute of Pathology and Molecular Pathology, Department XIII, Bundeswehrkrankenhaus Ulm, Ulm, Germany; 13 Institute of Neuropathology, Marburg University and University Medical Centre Marburg, Marburg, Germany; 14 Department of Pathology, School of Medicine and Health, Technical University Munich, Munich, Germany

**Keywords:** C-MYC, Neuroendocrine neoplasms, Neuroendocrine tumour, Neuroendocrine carcinoma

## Abstract

Neuroendocrine neoplasms (NEN) comprise well-differentiated neuroendocrine tumours (NET) and neuroendocrine carcinomas (NEC), whose distinction is clinically critical. Although c-MYC alterations have been implicated in NEC pathogenesis, c-MYC expression across NEC subtypes and anatomical sites, as well as in NET, remains incompletely defined. We analysed c-MYC immunohistochemically in 1380 resected NEN using the Immunoreactive Score (IRS: negative 0–1, weak 2–3, moderate 4–8, strong 9–12). Overall, c-MYC positivity (IRS ≥ 2) was observed in 13.3% of NEN. Expression was detected in 43% of NEC (164/381), including strong staining in 19.4%, whereas it was rare in NET and pulmonary carcinoids (20/999; 2%; p ≤ 0.001). Within NEC, c-MYC expression was enriched in LCNEC and MiNEN compared with SCNEC and Merkel cell carcinoma (p ≤ 0.001) and occurred more often in gastroenteropancreatic than in pulmonary NEC (57.6% vs. 37.3%; p ≤ 0.001). Among NET, G3 tumours showed the highest positivity rate (6/35; 17.1%), although this was significantly lower than in NEC (p ≤ 0.001), with strong expression observed in only one NET G3 (2.9%). No association between c-MYC expression and survival was identified in either NEC or NET. Our study confirms c-MYC expression as a common event in NEC and highlights differences across histological subtypes and anatomical sites, while demonstrating its absence in most low-proliferative NET. A subset of NET G3 tumours exhibits weak to moderate c-MYC expression at levels far below those seen in NEC, suggesting that strong c-MYC positivity may support a NEC classification in borderline cases but does not represent a definitive discriminatory marker.

**Supplementary Information:**

The online version contains supplementary material available at https://doi.org/10.1007/s12022-026-09935-x.

## Introduction

Neuroendocrine neoplasms (NEN) are broadly divided into well-differentiated neuroendocrine tumours (NET) and poorly differentiated neuroendocrine carcinomas (NEC). NEC also constitute the neuroendocrine component of most mixed neuroendocrine–non-neuroendocrine neoplasms (MiNEN), which are defined by the coexistence of a neuroendocrine and non-neuroendocrine component, each accounting for at least 30% of the neoplasm [1–5]. Accurate distinction between NET and NEC is clinically critical, particularly in morphologically ambiguous cases, as the two entities differ substantially in their biological behaviour, prognosis, and therapeutic management. NET show a variable, often prolonged clinical course, whereas NEC are characterised by rapid progression and poor outcomes [6–10].

Previous studies have described NET and NEC as generally distinct molecular entities [3, 11–13]. However, recent reports indicate that NEC-like transformation can occur during the disease course of NET [14]. Beyond frequent alterations in *TP53* and *RB1*, NEC show site-specific mutational patterns resembling those of conventional carcinomas arising in the same organ, whereas NET exhibit a distinct molecular spectrum with far fewer recurrent alterations [3, 12, 13, 15–19].

The (proto-)oncogene cellular myelocytomatosis (c-MYC) encodes a transcription factor involved in the regulation of cellular proliferation, survival, angiogenesis, metabolism, invasion and immune evasion [20–24]. C-MYC has been implicated as an oncogenic driver in gastric large cell NEC (LCNEC) and distinct subsets of pulmonary small cell NEC (SCNEC), while MYC alterations have also been described as relatively frequent events in gastrointestinal NEC [11, 25–29]. Recent advances in the development of specific inhibitors targeting the historically challenging c-MYC oncogene highlight its emerging therapeutic potential [24, 30–32].

However, the role of c-MYC across the broader spectrum of NEN remains insufficiently understood. It remains uncertain whether c-MYC expression is a universal feature of all NEC, varies between histological subtypes or differs across anatomical sites. Furthermore, it has yet to be determined whether c-MYC can also be frequently detected in NET and pulmonary carcinoids, or if its expression represents an exclusive event that generally distinguishes NEC from NET, potentially serving as a candidate biomarker for this distinction.

Therefore, we analysed c-MYC expression in a multicentric cohort of 1380 NEN from diverse anatomical sites, including matched metastatic lesions, and systematically compared expression patterns between NEC and NET overall, across their respective subtypes, and according to primary tumour location.

## Materials and Methods

### NEN Cohort Composition and Tumour Classification

A total of 1380 neuroendocrine neoplasms from 1205 patients were included in this multicentre study. Formalin-fixed, paraffin-embedded archival tumour tissue was retrieved from the Institutes of Pathology and Neuropathology of the University Medical Centres in Marburg, Mainz, Cologne, Erlangen–Nuremberg, Göttingen, Augsburg, Regensburg, and the Technical University of Munich, as well as from the Munich Municipal Hospital and the Bundeswehr Hospital (German Armed Forces Hospital) Ulm. Tumour samples from the participating centres (Marburg, Göttingen, Erlangen, Mainz, Ulm, and Cologne) were incorporated into tissue microarrays (TMA). Up to four representative cores per tumour were transferred using the TMA GrandMaster platform (Sysmex/3DHistech, Budapest, Hungary). Cases with insufficient tumour content or suboptimal fixation quality were excluded from this study.

The cohort had a median age at diagnosis of 61 years and included 515 female patients (42.7%) and 690 male patients (57.3%). Clinical and follow-up information was collected from institutional databases and regional cancer registries. Overall survival, defined as death from any cause, was available for 766 of 1205 patients, with follow-up censored at 120 months.

Prior to study inclusion, all NEN were re-evaluated and classified according to the current WHO classification for neuroendocrine neoplasms at their respective sites. Neoplasms classified as NET showed a differentiated neuroendocrine phenotype, characterised by monomorphic nuclei with finely stippled chromatin and an organoid growth architecture. These morphological features were accompanied by diffuse immunoreactivity for neuroendocrine markers combined with cytokeratin positivity. Proliferative activity assessed by the Ki-67 labelling fraction determined tumour grade (G1 < 3%, G2 3–20%, G3 > 20%) [1–5]. Pulmonary NET were classified as typical and atypical carcinoids according to the organ-specific WHO criteria. Typical carcinoids were defined by < 2 mitoses per 2 mm² and the absence of necrosis, whereas atypical carcinoids exhibited 2–10 mitoses per 2 mm² and/or focal tumour necrosis [2]. Additionally, we assessed the Ki-67 proliferation index following the grading principles established for gastroenteropancreatic NET to identify highly proliferative pulmonary carcinoids with proliferative activity comparable to NET G3. Neoplasms with high-grade morphology were classified as NEC when exhibiting brisk mitotic activity, necrotic areas, and solid growth. LCNEC was diagnosed in tumours composed of intermediate to large cells with vesicular chromatin and prominent nucleoli, whereas SCNEC was defined by small to medium-sized cells with scant cytoplasm, nuclear moulding, and hyperchromatic nuclei lacking visible nucleoli [1–5]. MiNEN, including combined pulmonary carcinomas, were defined by the coexistence of separate neuroendocrine and non-neuroendocrine carcinoma components, each comprising > 30% of the tumour. In this series, all MiNEN (including pulmonary combined carcinomas) consisted of a neuroendocrine carcinoma combined with either adenocarcinoma or squamous cell carcinoma [3].

### Immunohistochemical Assessment of c-MYC

Immunohistochemical staining was performed on tissue microarray sections using a BenchMARK XT/LT automated staining platform. c-MYC protein expression was detected using the monoclonal anti-c-MYC antibody Y69 (ab32072, Abcam, Cambridge, United Kingdom) at a dilution of 1:1000. Visualization was achieved using the OptiView DAB detection system (Roche Diagnostics, Mannheim, Germany). Prior to antibody incubation, heat-induced epitope retrieval was performed for 20 min in Tris/EDTA buffer (pH 9). Slides were subsequently counterstained with haematoxylin. Tissue from a Burkitt lymphoma harbouring a c-MYC::IGH fusion and showing strong c-MYC immunoreactivity served as the positive control, whereas omission of the primary antibody served as the negative control. All stainings were performed in a single laboratory (Institute of Pathology, Philipps University Marburg and University Medical Centre Marburg) using identical automated staining protocols to minimise inter-assay variability.

**Fig. 1:**
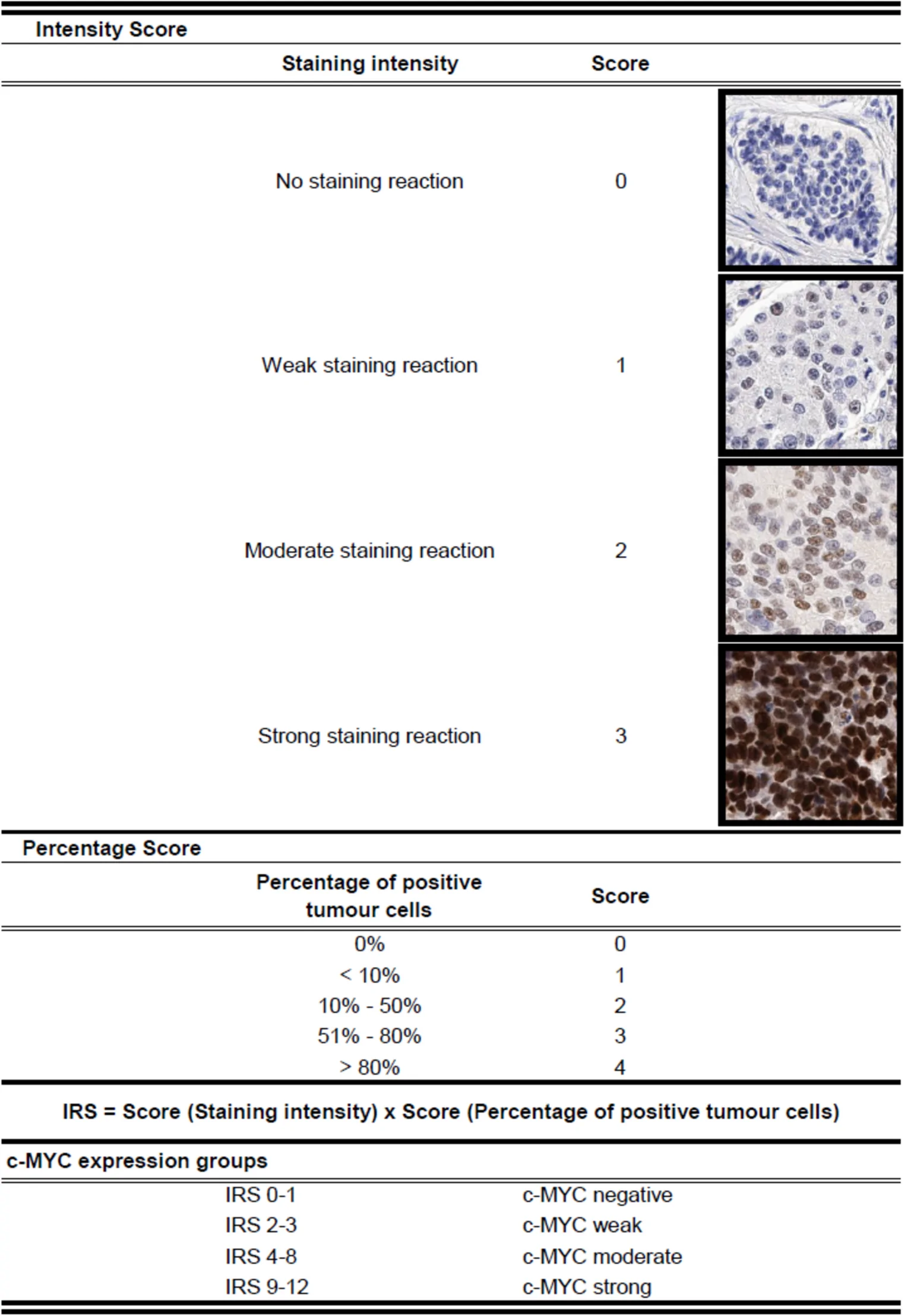
Classification of c-MYC expression according to the IRS. Immunoreactive Score (IRS) was derived by multiplying maximum staining intensity (0–3) by the fraction of immunoreactive tumour cells (0–4). According to IRS values, tumours were classified as c-MYC-negative (IRS ≤ 1), weak (IRS 2–3), moderate (IRS 4–8) or strong (IRS ≥ 9)

Nuclear c-MYC immunostaining was independently assessed by two pathologists (Moritz Jesinghaus, Maxime Schmitt), both blinded to clinicopathological data and patient outcome. The proportion of positively stained tumour cells was recorded across all available tissue microarray cores. Staining intensity was scored semi-quantitatively in four categories (absent to strong). An Immunoreactive Score (IRS) integrating staining intensity and percentage of positive cells was calculated. Based on IRS values, cases were categorized as negative (≤ 1), weak (2–3), moderate (4–8), or strong (≥ 9). An IRS ≥ 2 was predefined as the threshold for c-MYC positivity based on previous studies employing the same scoring methodology and comparable immunohistochemical assessment of nuclear biomarkers [33–35]. Details of the scoring system are provided in Fig. 1, and the receiver operating characteristic analysis (ROC) is shown in Supplementary Fig. 4.

For MiNEN, only c-MYC expression of the neuroendocrine component was evaluated on tissue microarrays. However, to address possible intra-tumoral heterogeneity of c-MYC expression between the two components, selected MiNEN (*n* = 18) with clear spatial separation of the neuroendocrine and non-neuroendocrine components were additionally assessed on whole-slide sections (for details see Supplementary Fig. 2).

Interobserver reproducibility was evaluated in 823 randomly selected tumours and demonstrated excellent agreement for both exact IRS categorization and dichotomized classification as positive versus negative cases (κ = 0.91 and κ = 0.98, respectively; *p* ≤ 0.001). Cases with discrepant scoring were reviewed jointly until consensus was achieved.

To evaluate the ability of c-MYC expression to discriminate NEC from NET G3, sensitivity, specificity, positive predictive value (PPV), negative predictive value (NPV), and overall accuracy were calculated for the predefined thresholds of c-MYC positivity (IRS ≥ 2) and strong c-MYC expression (IRS 9–12). Discriminatory performance across the entire IRS range was further assessed by receiver operating characteristic (ROC) analysis using the ordinal IRS as a continuous predictor. The area under the curve (AUC) was calculated with 95% confidence intervals (95% CI). The optimal cutoff was determined using the Youden index and corresponded to the predefined threshold for c-MYC positivity (IRS ≥ 2).

For an exploratory analysis of the biological context of c-MYC expression, immunohistochemical staining for p53 and RB1 was additionally performed on a randomly selected subset of 110 NEC from the original cohort. The subset comprised SCNEC (*n* = 49), LCNEC (*n* = 38), MiNEN (*n* = 12) and Merkel cell carcinoma (*n* = 11).

Immunohistochemical staining was performed on a Leica Bond III automated immunostainer (Leica Biosystems, Nussloch, Germany). Heat-induced epitope retrieval was carried out for 20 min using a citrate-based buffer (pH 6.0). p53 immunostaining was performed using the monoclonal mouse antibody DO-7 (NCL-p53-DO7, Leica Biosystems, Nussloch, Germany; dilution 1:50), and RB1 staining using the monoclonal mouse antibody G3-245 (554136, BD Pharmingen, Heidelberg, Germany; dilution 1:200). Antibody binding was visualized using the Bond Polymer Refine Detection System (Leica Biosystems, Nussloch, Germany), followed by haematoxylin counterstaining. High-grade serous ovarian carcinoma with diffuse p53 overexpression and normal appendix tissue with retained RB1 expression served as positive tissue controls.

p53 immunoreactivity was interpreted according to established criteria [36–40]. Diffuse strong nuclear staining in > 80% of tumour cells (overexpression pattern) or complete absence of nuclear staining in tumour cells in the presence of an internal positive control (null pattern) was classified as aberrant p53 expression, indicative of an underlying TP53 alteration. A heterogeneous pattern of variable nuclear staining intensity was considered consistent with wildtype p53 expression.

RB1 expression was considered retained when unequivocal nuclear staining was present in the tumour cells. Complete loss of nuclear RB1 expression in tumour cells, with preserved staining in internal non-neoplastic cells (endothelial cells and stromal cells) serving as a positive control, was interpreted as RB1 loss [3, 15, 41, 42].

Based on the combined p53 and RB1 immunophenotypes, four distinct molecular surrogate groups (p53^WT^/RB1^retained^, p53^aberrant^/RB1^retained^, p53^WT^/RB1^loss^, p53^aberrant^/RB1^loss^) were defined and correlated with c-MYC expression.

### Statistics

Statistical analyses were conducted using IBM SPSS Statistics version 29.0.2.0 (IBM Corp., Armonk, NY, USA). Chi-square (χ²) and Fisher’s exact tests (two-sided) were applied to assess associations between histological and clinicopathological features. Overall survival was estimated using Kaplan–Meier analysis, with group differences assessed by log-rank tests. To account for multiple comparisons, Bonferroni-adjusted p-values were additionally calculated. The principal findings remained unchanged after correction. Interobserver agreement was quantified using Cohen’s kappa statistic and interpreted according to the criteria proposed by Landis et al. [43]. Correlation of marker expression between different samples was assessed using Spearman’s rank correlation coefficient (ρ) for variables measured on an ordinal scale. Following Cohen’s conventions, a strong correlation was defined as ρ *≥ 0.5* [44]. Confidence intervals for the AUC were estimated using stratified bootstrap resampling (5000 iterations). For all statistical tests, *p-values ≤ 0.05* were regarded as statistically significant.

## Results

### Clinicopathological Features of the Overall NEN Cohort

**Fig. 2:**
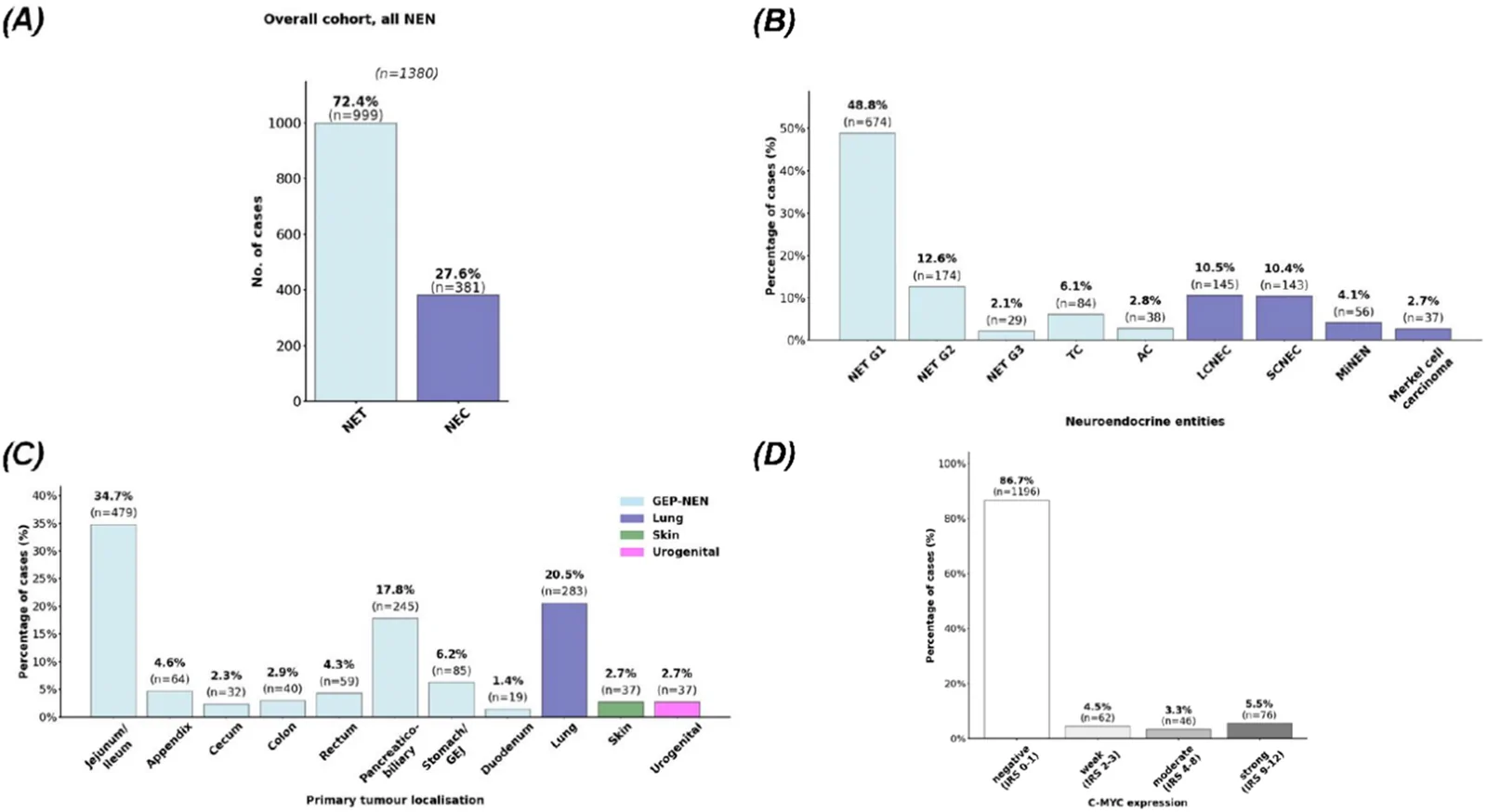

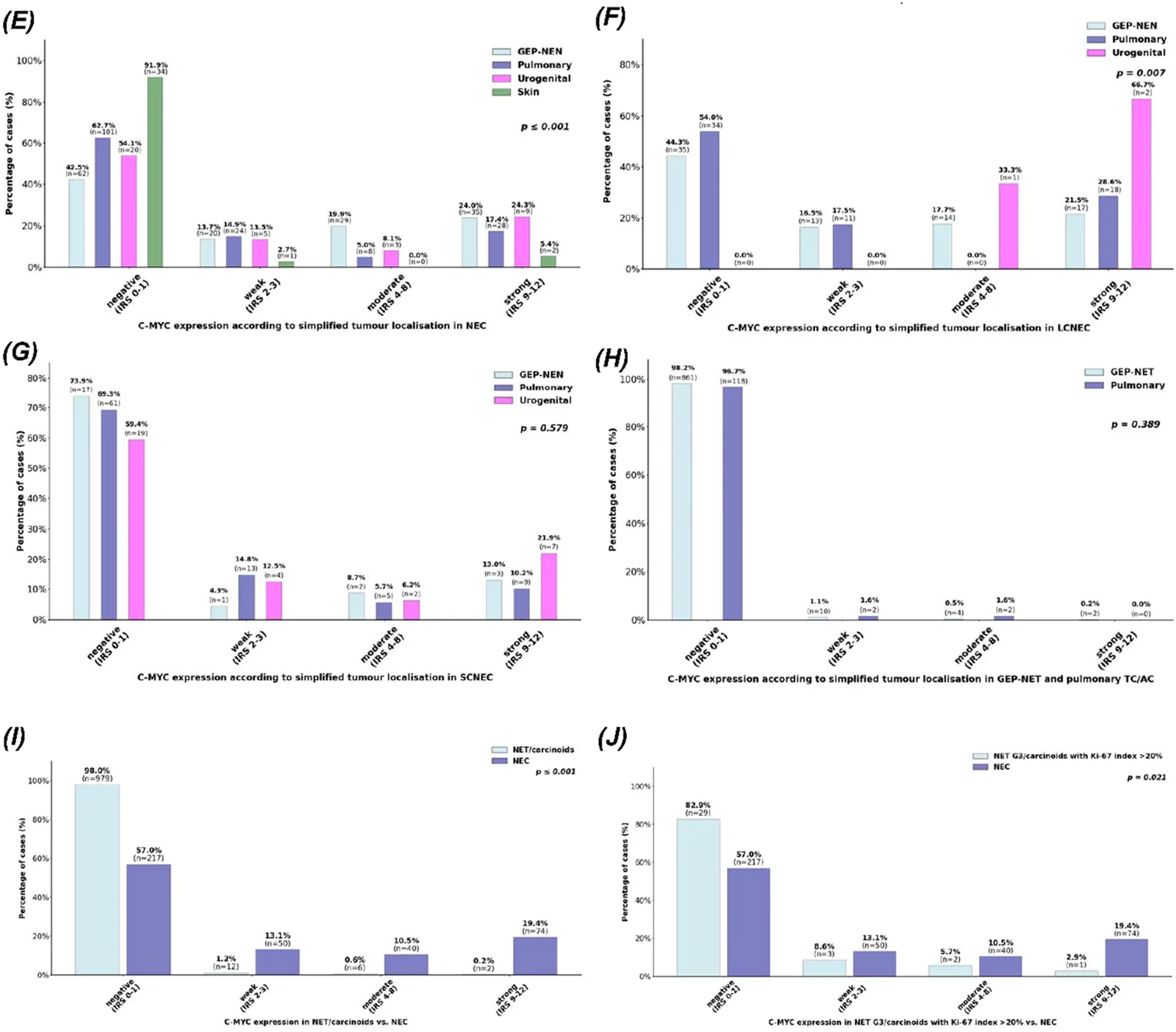
Overview of the study cohort and distribution patterns of c-MYC expression across neuroendocrine neoplasms. (**A**) Composition of the overall cohort showing the proportion of NET (including pulmonary typical and atypical carcinoids) and NEC (including MiNEN and Merkel cell carcinoma) among all analysed tumours (n = 1380). (**B**) Distribution of the individual neuroendocrine neoplasm subtypes within the complete cohort (n = 1380). (**C**) Anatomical origin of all investigated NEN cases (n = 1380). (**D**) Frequency of the different c-MYC IRS categories within the total cohort (n = 1380). (**E**) IRS-based c-MYC expression profiles in NEC according to simplified anatomical localisation groups (n = 381). (**F**) Distribution of c-MYC IRS groups in LCNEC by simplified localisation groups (n = 145) (**G**) Distribution of c-MYC IRS groups in SCNEC by simplified localisation groups (n = 143). (**H**) Distribution of c-MYC IRS groups in NET and pulmonary AC/TC (n = 999). (**I**) Comparison of c-MYC IRS groups between NET (including pulmonary TC/AC) and NEC (including MiNEN and MCC) (n = 1380). (**J**) Comparison of c-MYC IRS groups between NET G3/pulmonary carcinoids with Ki-67 > 20% and NEC (including MiNEN) (n = 416). c-MYC, cellular myelocytomatosis oncogene; NEN, neuroendocrine neoplasm; NET, neuroendocrine tumour; NEC, neuroendocrine carcinoma, LCNEC, large cell neuroendocrine carcinoma; SCNEC, small cell neuroendocrine carcinoma; MiNEN, mixed neuroendocrine-non-neuroendocrine carcinoma; TC, typical carcinoid; AC, atypical carcinoid; GEP, gastroenteropancreatic; MCC, Merkel cell carcinoma; IRS, Immunoreactive Score

The cohort comprised 1380 NEN samples from 1205 patients including 999 (72.4%) NET (including pulmonary carcinoids) and 381 (27.6%) NEC (Fig. 2A; Table 1). Among NEC, 143 (37.5%) were diagnosed as SCNEC, 145 (38.1%) as LCNEC, 56 (14.7%) as MiNEN and 37 (9.7%) as Merkel cell carcinoma. NET grading revealed 674 (67.5%) NET G1, 174 (17.4%) NET G2, and 29 (3.3%) NET G3, in addition to 84 (8.4%) pulmonary TC and 38 (3.8%) pulmonary AC (Fig. 2B). Primary sites included 283 (20.5%) pulmonary NEN (122/43.1% TC/AC, 161/56.9% NEC), 1023 (74.1%) gastroenteropancreatic (GEP)-NEN (877/85.7% NET, 146/14.3% NEC), 37 (2.7%) cutaneous Merkel cell carcinomas and 37 (2.7%) urogenital NEC (Fig. 2C).

**Table 1:** Comprehensive clinicopathological overview of the neuroendocrine neoplasm cohort, including histological classification, simplified anatomical grouping, and detailed site-specific tumour distribution

			c-MYC expression groups			
			c-MYC negative (IRS0-1)	c-MYC weak (IRS2-3)	c-MYC moderate (IRS4-8)	c-MYC strong (IRS9-12)
		Overall *n* (%)	1196 (86.7%)	62 (4.5%)	46 (3.3%)	76 (5.5%)
Tumour classification						
NET		999 (72.4%)	979 (98.0%)	12 (1.2%)	6 (0.6%)	2 (0.2%)
	NET G1	674 (67.5%)	665 (98.7%)	6 (0.9%)	2 (0.3%)	1 (0.1%)
	NET G2	174 (17.4%)	173 (99.4%)	1 (0.6%)	0 (0.0%)	0 (0.0%)
	NET G3	29 (3.3%)	23 (79.3%)	3 (10.3%)	2 (6.9%)	1 (3.4%)
	TC	84 (8.4%)	82 (97.6%)	1 (1.2%)	1 (1.2%)	0 (0.0%)
	AC	38 (3.8%)	36 (94.7%)	1 (2.6%)	1 (2.6%)	0 (0.0%)
NEC		381 (27.6%)	217 (57.0%)	50 (13.1%)	40 (10.5%)	74 (19.4%)
	LCNEC	145 (38.1%)	69 (47.6%)	24 (16.6%)	15 (10.3%)	37 (25.5%)
	SCNEC	143 (37.5%)	97 (67.8%)	18 (12.6%)	9 (6.3%)	19 (13.3%)
	MiNEN	56 (14.7%)	17 (30.4%)	7 (12.5%)	16 (28.6%)	16 (28.6%)
	MCC	37 (9.7%)	34 (91.9%)	1 (2.7%)	0 (0.0%)	2 (5.4%)
Simplified tumour localisation						
NET		999 (72.4%)	979 (98.0%)	12 (1.2%)	6 (0.6%)	2 (0.2%)
	*GEP-NET*	*877 (87.6%)*	*861 (98.2%)*	*10 (1.1%)*	*4 (0.5%)*	*2 (0.2%)*
		*674 (76.9%)*	*665 (98.7%)*	*6 (0.9%)*	*2 (0.3%)*	*1 (0.1%)*
		*174 (19.8%)*	*173 (99.4%)*	*1 (0.6%)*	*0 (0.0%)*	*0 (0.0%)*
		*29 (3.3%)*	*23 (79.3%)*	*3 (10.3%)*	*2 (6.9%)*	*1 (3.4%)*
	*pulmonary carcinoids*	*122 (12.2%)*	*118 (96.7%)*	*2 (1.6%)*	*2 (1.6%)*	*0 (0.0%)*
		*84 (8.4%)*	*82 (97.6%)*	*1 (1.2%)*	*1 (1.2%)*	*0 (0.0%)*
		*38 (3.8%)*	*36 (94.7%)*	*1 (2.6%)*	*1 (2.6%)*	*0 (0.0%)*
NEC		381 (27.6%)	217 (57.0%)	50 (13.1%)	40 (10.5%)	74 (19.4%)
	*pulmonary NEC*	*161 (42.3%)*	*101 (62.7%)*	*24 (14.9%)*	*8 (5.0%)*	*28 (17.4%)*
		*88 (54.7%)*	*61 (69.3%)*	*13 (14.8%)*	*5 (5.7%)*	*9 (10.2%)*
		*63 (39.1%)*	*34 (54.0%)*	*11 (17.5%)*	*0 (0.0%)*	*18 (28.6%)*
		*10 (6.2%)*	*6 (60.0%)*	*0 (0.0%)*	*3 (30.0%)*	*1 (10.0%)*
	*GEP-NEC*	*146 (38.3%)*	*62 (42.5%)*	*20 (13.7%)*	*29 (19.9%)*	*35 (24.0%)*
		*23 (15.8%)*	*17 (73.9%)*	*1 (4.3%)*	*2 (8.7%)*	*3 (13.0%)*
		*79 (54.1%)*	*35 (44.3%)*	*13 (16.5%)*	*14 (17.7%)*	*17 (21.5%)*
		*44 (30.1%)*	*10 (22.7%)*	*6 (13.6%)*	*13 (29.5%)*	*15 (34.1%)*
	*urogenital NEC*	*37 (9.7%)*	*20 (54.1%)*	*5 (13.5%)*	*3 (8.1%)*	*9 (24.3%)*
		*32 (86.5%)*	*19 (59.4%)*	*4 (12.5%)*	*2 (6.3%)*	*7 (21.9%)*
		*3 (8.1%)*	*0 (0.0%)*	*0 (0.0%)*	*1 (33.3%)*	*2 (66.7%)*
		*2 (5.4%)*	*1 (50.0%)*	*1 (50.0%)*	*0 (0.0%)*	*0 (0.0%)*
	*Skin-NEC*	*37 (9.7%)*	*34 (91.9%)*	*1 (2.7%)*	*0 (0.0%)*	*2 (5.4%)*

### c-MYC Expression Across the Entire NEN Cohort

**Fig. 3:**
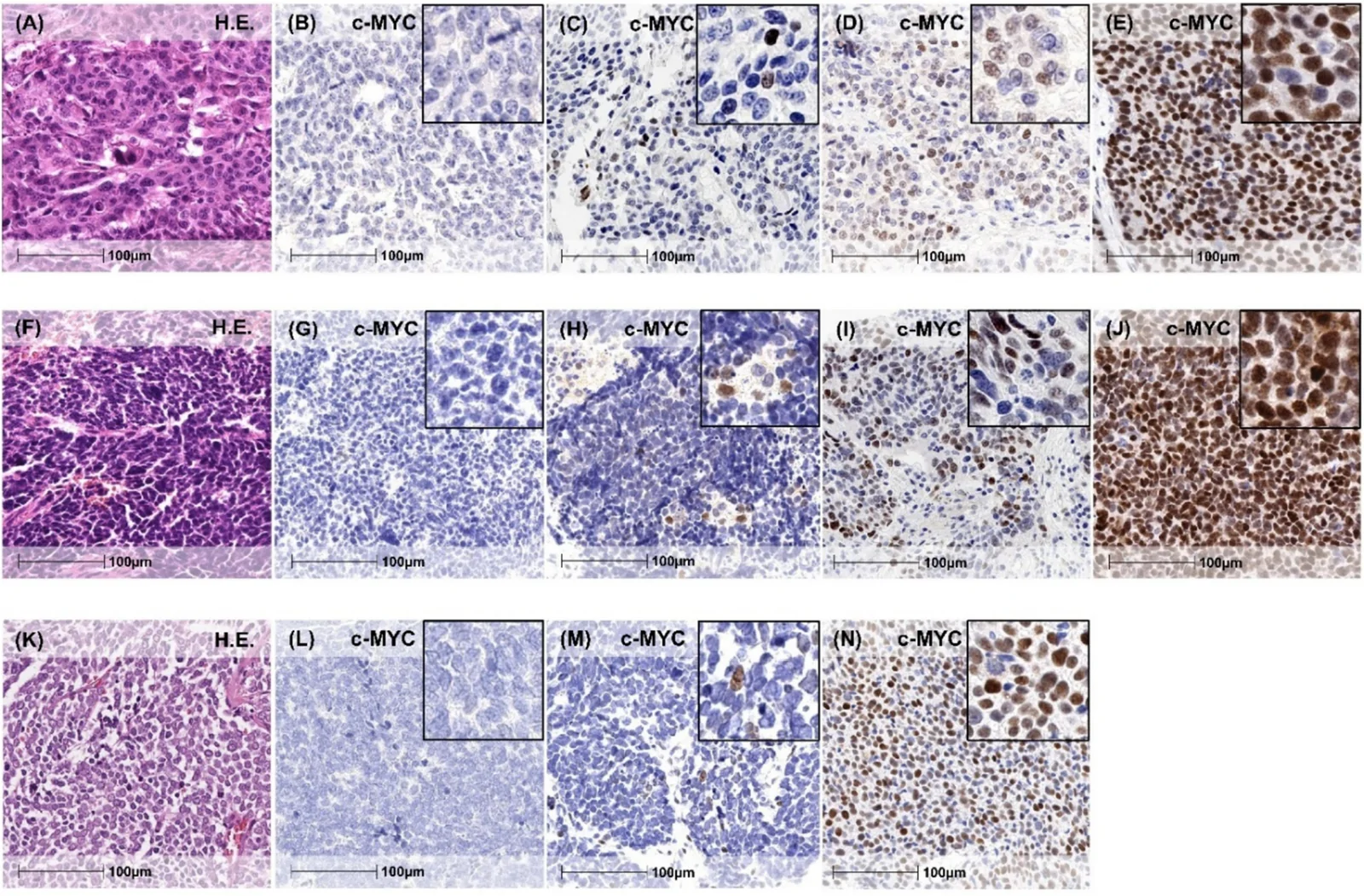
Expression of c-MYC in NEC and Merkel cell carcinoma. (**A**-**E**) Representative cases of large cell neuroendocrine carcinoma (LCNEC). (**A**) Rectal LCNEC shown in haematoxylin and eosin staining (H.E.). (**B**) Rectal LCNEC lacking detectable c-MYC expression (IRS 0). (**C**) Colonic LCNEC with weak c-MYC expression (IRS 3; up to strong staining intensity in < 10% of tumour cells). (**D**) Gastric LCNEC demonstrating moderate nuclear c-MYC expression (IRS 6; up to moderate staining intensity in > 50% of tumour cells). (**E**) Pulmonary LCNEC with strong c-MYC expression (IRS 12; strong staining intensity in > 80% of tumour cells). (**F**-**J**) Representative cases of small cell neuroendocrine carcinoma (SCNEC). (**F**) Pulmonary SCNEC shown in haematoxylin and eosin staining (H.E.) (**G**) Pulmonary SCNEC without detectable nuclear c-MYC expression (IRS 0). (H) Pulmonary SCNEC with weak c-MYC expression (IRS 3; up to strong staining intensity in < 10% of tumour cells). (I) Pulmonary SCNEC with moderate c-MYC expression (IRS 6; strong staining intensity in > 10% of tumour cells). (**J**) Vaginal SCNEC with strong c-MYC expression (IRS 12; strong staining intensity in > 80% of tumour cells). (K-N) Representative cases of Merkel cell carcinomas. (**K**) Merkel cell carcinoma shown in haematoxylin and eosin staining (H.E.). (**L**) Merkel cell carcinoma with complete absence of c-MYC expression (IRS 0). (M) Merkel cell carcinoma with weak c-MYC expression (IRS 3; up to strong expression intensity in < 10% of carcinoma cells). (**N**) Merkel cell carcinoma with strong c-MYC expression (IRS 9; up to strong staining intensity in > 50% of carcinoma cells). Overview: 40x magnification, Inlay: 100x magnification. H.E., haematoxylin and eosin; c-MYC, cellular myelocytomatosis oncogene; IRS, Immunoreactive Score; NEC, neuroendocrine carcinoma; LCNEC, large cell neuroendocrine carcinoma; SCNEC, small cell neuroendocrine carcinoma; MCC, Merkel cell carcinoma

**Fig. 4:**
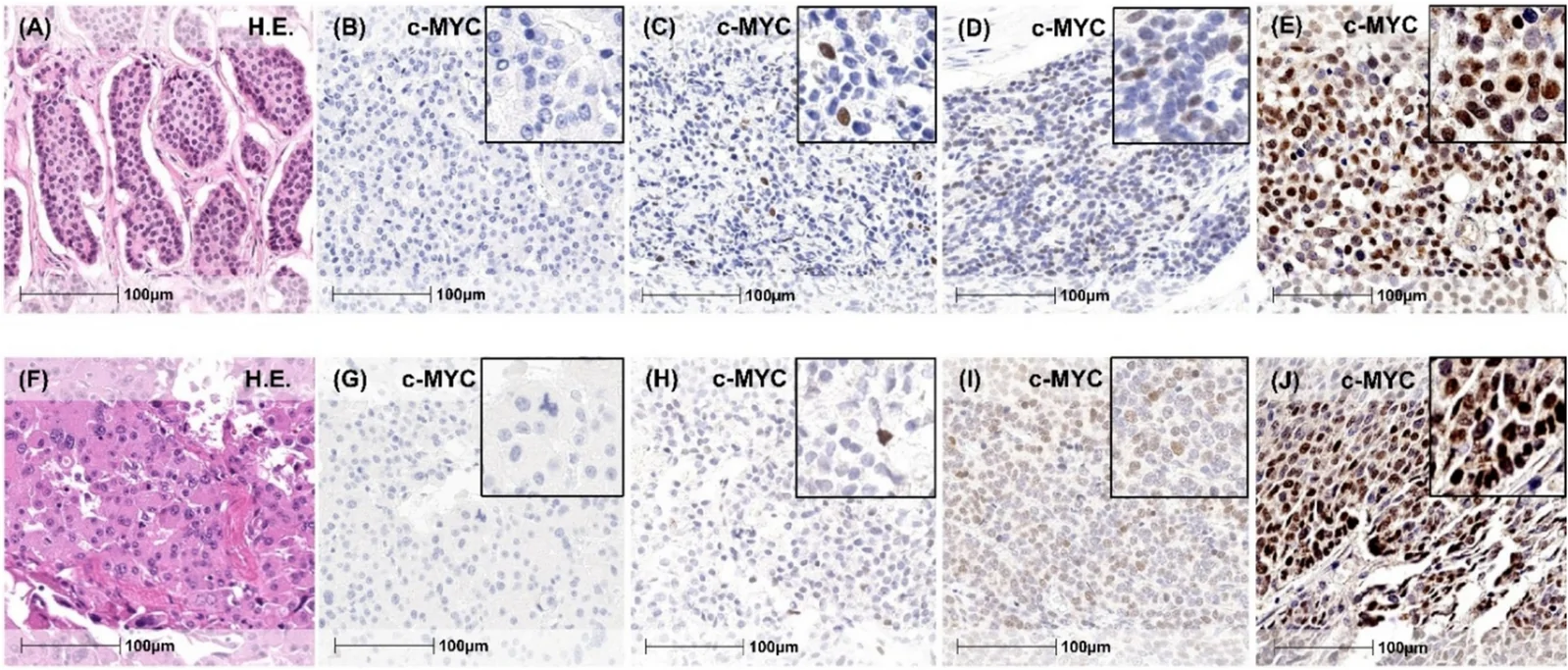
Expression of c-MYC in NET. (**A**-**E**) Representative cases of NET G1 and G2. (**A**) Ileal NET (G1) shown in haematoxylin and eosin staining (H.E.). (**B**) Pancreatic NET (G2) lacking c-MYC expression (IRS 0). (**C**) Pancreatic NET (G1) with weak c-MYC expression (IRS 3; up to strong nuclear staining reaction in < 10% of tumour cells). (**D**) Gastric NET (G1) with moderate c-MYC expression (IRS 4; moderate staining intensity in > 10% of tumour cells). (**E**) Cecal NET (G1) with strong c-MYC expression (IRS 12; strong staining intensity in > 80% of tumour cells). (**F**-**J**) Representative cases of NET G3 (**F**) Pancreatic NET (G3) shown in haematoxylin and eosin staining (H.E.). (**G**) Pancreatic NET (G3) with complete absence of c-MYC expression (IRS 0). (H) Pancreatic NET (G3) with weak c-MYC expression (IRS 3; up to strong expression intensity in < 10% of tumour cells). (**I**) Gastric NET (G3) with moderate c-MYC expression (IRS 6; moderate staining intensity in > 50% of tumour cells). (**J**) Pancreatic NET (G3) with strong c-MYC expression (IRS 12; strong staining intensity in > 80% of tumour cells). Overview: 40x magnification, Inlay: 100x magnification. H.E., haematoxylin and eosin; c-MYC, cellular myelocytomatosis oncogene; IRS, Immunoreactive Score; NET, neuroendocrine tumour

Among all 1380 analysed NEN, detectable c-MYC immunoreactivity was identified in 184/1380 (13.3%) cases. According to the IRS classification, 62 cases (4.5%) showed weak expression (IRS 2–3), 46 cases (3.3%) moderate expression (IRS 4–8), and 76 cases (5.5%) strong expression (IRS 9–12), whereas 1196/1380 (86.7%) cases showed no detectable c-MYC expression (Fig. 2D). IRS categories are illustrated in Fig. 3 for NEC and Merkel cell carcinoma and in Fig. 4 for NET.

### c-MYC Expression in NEC and MiNEN

Within the NEC subgroup, c-MYC positivity was observed in 164/381 cases (43.0%; *p* ≤ 0.001), including 50 with weak (13.1%), 40 (10.5%) with moderate and 74 (19.4%) with strong expression according to the IRS score (*p* ≤ 0.001). LCNEC (76/145, 52.4%) and MiNEN (39/56, 69.6%) exhibited the highest proportion of c-MYC-positive cases compared with SCNEC (46/143, 32.2%) and Merkel cell carcinoma (3/37, 8.1%) when considering expression of any degree (*p* ≤ 0.001). Strong c-MYC expression was most frequent in LCNEC (37/145, 25.5%) and MiNEN (16/56, 28.6%), compared with SCNEC (19/143, 13.3%) (*p* ≤ 0.001).

In site-specific analyses, GEP-NEC showed the highest frequency of c-MYC positivity (84/146, 57.6%) as well as enrichment for moderate to strong expression (64/146, 43.9%) compared with urogenital (12/37, 32.4%) and pulmonary (36/161, 22.4%) NEC (*p* ≤ 0.001) (Fig. 2E).

Additional whole-slide evaluation of MiNEN (*n* = 18) demonstrated c-MYC immunoreactivity in both tumour compartments in the majority of cases (16/18, 88.8%). In most tumours, expression levels were either comparable between the two components (10/18, 55.5%) or higher in the neuroendocrine compartment (6/18, 33.3%). Detailed findings are summarised in Supplementary Fig. 2.

### Association of c-MYC Expression with p53 and RB1 Immunophenotype in NEC

To further explore the biological context of c-MYC expression, p53 and RB1 immunohistochemistry was performed in a randomly selected subset of 110 NEC (including SCNEC, LCNEC, MiNEN and Merkel cell carcinoma). Aberrant p53 expression was observed in 86 NEC (78.2%), whereas loss of RB1 expression was present in 74 NEC (67.3%). No association between c-MYC positivity and p53 immunophenotype was observed, either across individual IRS groups (*p* = 0.857) or after dichotomization of c-MYC expression as positive vs. negative (*p* = 0.493). In contrast, retained RB1 expression was significantly enriched among c-MYC positive tumours (IRS ≥ 2) (*p* = 0.01) and associated with strong c-MYC expression (IRS ≥ 9) (41.7% RB1^retained^ vs. 17.6% RB1^loss^; *p* = 0.006). Analysis of the combined p53/RB1 immunophenotypes revealed a non-significant trend towards differential c-MYC expression (*p* = 0.063). Strong c-MYC expression was most frequently observed in tumours with retained RB1 expression, irrespective of p53 status (40.0% p53^WT^/RB1^retained^ and 42.9% p53^aberrant^/RB1^retained^ tumours). In contrast, no case with the p53^WT^/RB1^loss^ phenotype exhibited strong c-MYC expression, whereas only 20.0% of tumours with combined p53 aberration and RB1 loss showed strong c-MYC expression. Detailed results are provided in Supplementary Table 1.

### c-MYC Expression in NET and Pulmonary Carcinoids

c-MYC expression was rare in NET, being detected in only 20/999 (2.0%) tumours. Most positive c-MYC-positive NET showed weak (12/999, 1.2%) or moderate (6/999, 0.6%) expression, while 979/999 (98.0%) tumours were completely c-MYC-negative. Strong c-MYC expression was observed in only two NET (0.2%; *p* ≤ 0.001). No statistically significant differences in expression frequency were detected between gastroenteropancreatic NET and pulmonary carcinoids (*p* = 0.389) (Fig. 2H).

### c-MYC Expression in NET vs. NEC

Overall c-MYC positivity was significantly more common in NEC (including MiNEN and MCC) than in NET/TC/AC (164/381, 43.0% vs. 20/999, 2.0%; *p* ≤ 0.001). This was particularly evident for moderate to strong IRS scores, which were also markedly enriched in NEC (114/381, 29.9% vs. 8/999, 0.8% in NET; *p* ≤ 0.001).

### c-MYC Expression in NET G3 and Carcinoids with Ki-67 index > 20% Compared with NEC

When well-differentiated NEN were subgrouped according to their Ki-67 index, NET G3 and carcinoids with Ki-67 > 20% showed a significantly higher overall c-MYC expression (positive vs. negative) compared with low-proliferative tumours (NET G1/2 and carcinoids with Ki-67 ≤ 20%) (6/35, 17.1% vs. 14/964, 1.5%; *p* ≤ 0.001). However, stratification by IRS categories revealed that c-MYC expression in NET G3 and carcinoids with Ki-67 > 20% was predominantly weak (3/35, 8.6%) or moderate (2/35, 5.7%), with only a single case showing strong expression (1/35, 2.9%; *p* ≤ 0.001). Comparative analysis of NET G3/carcinoids with Ki-67 > 20% and NEC further demonstrated a markedly higher overall c-MYC expression in NEC (164/381, 43.0%) than in highly proliferative NET (6/35, 17.1%; *p* = 0.003). Consistently, strong c-MYC expression was considerably more frequent in NEC (74/381, 19.4%) than in highly proliferative NET (1/35, 2.9%; *p* = 0.021) (Fig. 2J).

### Diagnostic Performance of c-MYC Expression in Discriminating NEC vs. NET G3

To assess the diagnostic utility of c-MYC immunohistochemistry for distinguishing NEC from NET G3 in diagnostically challenging cases, c-MYC positivity (IRS ≥ 2) demonstrated a sensitivity of 43.0%, a specificity of 79.3%, a positive predictive value (PPV) of 96.5%, a negative predictive value (NPV) of 9.6%, and an overall accuracy of 45.6% (Supplementary Fig. 4A). Receiver operating characteristic analysis using the ordinal c-MYC Immunoreactive Score (IRS) yielded an area under the curve (AUC) of 0.624 (95% CI, 0.549–0.693; *p* = 0.001), indicating modest discriminatory performance between NEC and NET G3. The optimal cutoff according to the Youden index was IRS ≥ 2, corresponding to the predefined threshold for c-MYC positivity (Supplementary Fig. 4B). In contrast, restricting positivity to strong c-MYC expression (IRS 9–12) substantially increased specificity (96.6%) and positive predictive value (98.7%), albeit at the expense of sensitivity, supporting the use of strong c-MYC expression as a highly specific ancillary marker for the diagnosis of NEC (Supplementary Fig. 4C).

### Prognostic Implications of c-MYC Expression

Across the entire cohort, c-MYC-positive NEN were associated with shorter overall survival compared with c-MYC-negative neoplasms (median OS: 46 vs. 94 months; *p* ≤ 0.001). Significant survival differences were also observed across IRS expression categories (*p* ≤ 0.001). However, these findings largely reflected the well-established prognostic disparity between NEC and NET, with NEC showing substantially inferior survival compared with NET (median OS: 35 vs. 103 months; *p* ≤ 0.001).

When analysed separately within NET and NEC subgroups, c-MYC expression did not demonstrate independent prognostic significance (NET: *p* = 0.15; NEC: *p* = 0.858; Supplementary Fig. 3). Similar results were obtained after further stratification by anatomical site (data not shown).

## Discussion

c-MYC is a well-established master regulator of oncogenesis across multiple tumour entities. Recent advances in the development of targeted inhibitors against this historically challenging oncogene underscore its emerging therapeutic potential [24, 30–32]. Although previous studies have identified c-MYC activation as a key event in gastric LCNEC and subsets of pulmonary SCNEC [11, 25–29], a comprehensive characterisation of c-MYC expression across the full spectrum of NEN has been lacking. In this study, we provide a systematic analysis of c-MYC expression in a large, multicentre cohort of 1380 NEN, encompassing all major morphological subtypes and anatomical sites. c-MYC expression was identified as a relatively frequent feature of NEC, detected in 43% of cases, including 19.4% with strong positivity. In contrast, c-MYC expression was rare in GEP-NET and pulmonary carcinoids. Although NET G3 (including pulmonary carcinoids with a Ki-67 index above 20%) showed higher expression levels than NET G1/G2, staining remained predominantly weak to moderate and was significantly lower than in NEC. These findings identify c-MYC expression as a feature of a substantial subset of NEC and provide a rationale for further investigating MYC-directed therapeutic strategies in these highly aggressive neoplasms. Within NEC, c-MYC expression varied significantly by histological subtype and anatomical site. LCNEC and MiNEN showed the highest rates of c-MYC positivity and were enriched for higher IRS categories compared with SCNEC and Merkel cell carcinoma. These findings are consistent with previous reports of frequent c-MYC alterations in LCNEC and MiNEN and further suggest a potential role for c-MYC in their pathogenesis [25, 45–47]. In MiNEN, c-MYC expression was largely concordant between the neuroendocrine and non-neuroendocrine components, suggesting that c-MYC may act as an oncogenic driver in both components. In several cases, however, individual IRS values differed between the two components, with stronger staining observed in the neuroendocrine component.

To further explore the biological context of c-MYC expression, we analysed its relationship with the two hallmark tumour suppressor proteins p53 and RB1 in an exploratory subset of 110 NEC. While c-MYC expression was not associated with the p53 immunophenotype, a significant association was observed with retained RB1 expression. Interestingly, strong c-MYC expression was enriched in RB1-retained tumours irrespective of p53 status, whereas strong c-MYC expression was comparatively uncommon in NEC with combined aberrant p53 expression and RB1 loss. This finding suggests that c-MYC expression in NEC is not merely a surrogate of the canonical TP53/RB1-deficient phenotype. Instead, c-MYC may define a biologically distinct subset of NEC in which c-MYC expression occurs despite preserved RB1 protein expression. This observation is biologically plausible, as MYC activation can promote cell-cycle progression through multiple mechanisms beyond RB1 inactivation [29, 48–51]. Conversely, MYC overexpression is not an obligatory consequence of RB1 loss, indicating that these alterations may represent partially independent oncogenic events during NEC evolution. Nevertheless, these findings should be interpreted with caution, as RB1 and p53 status were inferred from immunohistochemical surrogate markers rather than molecular analyses, and the exploratory subgroup was of limited size. Future studies integrating genomic assessment of RB1 and TP53 together with MYC copy number and expression analyses will be required to clarify the biological interaction between these pathways.

The enrichment of c-MYC expression in NEC also raises the clinically relevant question of whether c-MYC immunohistochemistry may aid in distinguishing NET G3 from NEC. Overall, c-MYC expression was significantly lower in NET G3 (including pulmonary carcinoids with a Ki-67 index above 20%) than in NEC (*p* = 0.003). Moreover, expression in these tumours was almost exclusively weak to moderate, whereas strong staining was identified in only a single NET G3. Although ROC analysis identified IRS ≥ 2 as the statistically optimal threshold according to the Youden index, this cutoff primarily maximizes the balance between sensitivity and specificity. In routine pathology, however, a highly specific ancillary marker is often more valuable than a balanced screening marker when distinguishing morphologically challenging NET G3 from NEC. Therefore, a threshold for strong c-MYC expression (IRS 9–12), despite its lower sensitivity, may be of greater practical diagnostic relevance because of its excellent specificity and very high positive predictive value. Consequently, strong c-MYC expression may provide supportive evidence for a diagnosis of NEC in morphologically ambiguous cases. However, c-MYC is not a universal discriminator. A substantial proportion of NEC lacked c-MYC expression, indicating that MYC-independent oncogenic pathways also exist. Conversely, weak to moderate staining was observed in approximately 14% of NET G3. Therefore, c-MYC immunohistochemistry should always be interpreted in conjunction with morphology and established diagnostic markers rather than as a stand-alone test. Interestingly, c-MYC expression was significantly higher in NET G3 than in NET G1/G2. This observation may reflect increasing molecular complexity during tumour progression, although the underlying mechanisms remain unknown. Whether secondary MYC activation contributes to disease progression or the acquisition of NEC-like features during tumour evolution warrants further investigation [14, 52].

Several limitations of our study should be considered. First, the retrospective design and predominant use of tissue microarrays in this large multicentre cohort may introduce sampling bias. Although all immunohistochemical analyses were performed centrally using standardized protocols, variability in fixation and tissue processing across the contributing institutions cannot be completely excluded. Second, c-MYC status was assessed exclusively at the protein level. Therefore, the molecular mechanisms underlying c-MYC overexpression could not be addressed directly. Future studies integrating genomic, transcriptomic, and proteomic analyses will be required to further elucidate the molecular mechanisms underlying c-MYC overexpression in neuroendocrine neoplasms. Third, owing to their rarity, only 29 NET G3 and six pulmonary carcinoids with Ki-67 indices > 20% were included, limiting conclusions regarding the discriminatory value of c-MYC for differentiating NET G3 from NEC. This potential diagnostic utility should therefore be further evaluated in larger NET G3 cohorts. Finally, our study did not include longitudinal samples from NET undergoing NEC-like transformation, a setting in which the potential role of c-MYC warrants further investigation.

In summary, c-MYC expression is frequently detectable in NEC, with marked variability across histological subtypes and anatomical sites, highlighting a subset of NEC that may warrant further investigation in the context of MYC-directed therapeutic strategies. Strong c-MYC expression appears almost exclusively in NEC and may provide useful diagnostic support in distinguishing NEC from NET G3 in morphologically challenging cases. From a practical diagnostic perspective, c-MYC immunohistochemistry should be regarded as an ancillary rather than a stand-alone diagnostic marker, and interpreted in conjunction with morphology, proliferation rate, and established neuroendocrine markers.

Future molecular studies integrating genomic and transcriptomic analyses are warranted to further elucidate the biological basis of c-MYC overexpression in neuroendocrine neoplasms.

## Supplementary Information

Below is the link to the electronic supplementary material.

Supplementary Figure 1 (JPEG 3.47 MB)

Supplementary Figure 2 (JPEG 6.96 MB)

Supplementary Figure 3 (JPEG 3.32 MB)

Supplementary Figure 4 (JPEG 1.13 MB)

Supplementary Table 1 (PDF 106 KB)

## Data Availability

Tissue samples and associated datasets generated and analysed during the current study are archived at the Institute of Pathology, Philipps University Marburg and University Medical Centre Marburg, Germany. Access to these materials may be granted by the corresponding author upon reasonable request and in accordance with institutional regulations.

## Declarations

### Regarding the use of AI-assisted technologies

During manuscript preparation, the authors used ChatGPT (OpenAI Ireland Ltd., Dublin, Ireland) and Perplexity AI (Perplexity AI Inc., San Francisco, CA, USA) exclusively for linguistic support and spelling correction. All generated suggestions were carefully reviewed, revised where necessary, and fully verified by the authors, who retain complete responsibility for the final content of the manuscript.
