# Supplementary material for: Divergent c-MYC Expression Patterns in NET and NEC: Insights from a Multicentre Cohort of 1380 Neuroendocrine Neoplasms
Source: Endocr Pathol. 2026 Sep 12;37(1):39. doi: 10.1007/s12022-026-09935-x (PMC13570926; doi:10.1007/s12022-026-09935-x)
Supplement: Supplementary file 1 [file 12022_2026_9935_MOESM4_ESM.pdf]

Supplementary Table 1

(A)	Overall n (%)	c-MYC negative (IRS 0–1)	c-MYC positive (IRS ≥2)	*p-value*
	110 (100 %)	*62 (56.4 %)*	*48 (43.6%)*	
**p53**				
Wildtype (WT)	*24 (21.8 %)*	15 (62.5%)	9 (37.5%)	*0.493*
Aberrant expression (aberrant)	*86 (78.2 %)*	47 (54.7%)	39 (45.3%)	
**RB1**				
Loss	*74 (67.3%)*	48 (64.9%)	26 (35.1%)	*0.010*
Retained expression	*36 (32.7%)*	14 (38.9%)	22 (61.1%)	
**Combined p53/RB1 phenotype**				
p53 WT/ RB1 retained	*15 (13.6%)*	7 (46.7%)	8 (53.3%)	*0.021*
p53 aberrant/ RB1 retained	*21 (19.1%)*	7 (33.3%)	14 (66.7%)	
p53 WT/ RB1 loss	*9 (8.2%)*	8 (88.9%)	1 (11.1%)	
p53 aberrant/ RB1 loss	*65 (59.1%)*	40 (61.5%)	25 (38.5%)	

(B)	Overall n (%)	c-MYC negative (IRS 0–1)	c-MYC weak (IRS 2-3)	c-MYC moderate (IRS 5-8)	c-MYC strong (IRS 9-12)	*p-value*
	110 (100 %)	*62 (56.4 %)*	*14 (12.7 %)*	*6 (5.5 %)*	*28 (25.5 %)*	
**p53**						
Wildtype (WT)	*24 (21.8 %)*	15 (62.5 %)	2 (8.3 %)	1 (4.2 %)	6 (21.4 %)	*0.857*
Aberrant expression (aberrant)	*86 (78.2 %)*	47 (54.7 %)	12 (14.0 %)	5 (5.8 %)	22 (25.6 %)	
**RB1**						
Loss	*74 (67.3%)*	48 (64.9%)	11 (14.9 %)	2 (2.7 %)	13 (17.6 %)	*0.006*
Retained expression	*36 (32.7%)*	14 (38.9%)	3 (8.3 %)	4 (11.1 %)	15 (41.7 %)	
**Combined p53/RB1 phenotype**						
p53 WT/ RB1 retained	*15 (13.6%)*	7 (46.7%)	1 (6.7 %)	1 (6.7 %)	6 (40.0 %)	*0.063*
p53 aberrant/ RB1 retained	*21 (19.1%)*	7 (33.3%)	2 (9.5 %)	3 (50.0 %)	9 (42.9 %)	
p53 WT/ RB1 loss	*9 (8.2%)*	8 (88.9%)	1 (11.1 %)	0 (0.0 %)	0 (0.0 %)	
p53 aberrant/ RB1 loss	*65 (59.1%)*	40 (61.5%)	10 (15.4 %)	2 (33.3 %)	13 (20.0 %)	
